# Interaction of pig manure-derived dissolved organic matter with soil affects sorption of sulfadiazine, caffeine and atenolol pharmaceuticals

**DOI:** 10.1007/s10653-021-00904-3

**Published:** 2021-04-15

**Authors:** Wei Zhang, Xiangyu Tang, Sören Thiele-Bruhn

**Affiliations:** 1 Soil Science, University of Trier, Behringstraße 21, 54296 Trier, Germany; 2 Present Address: School of Tourism and Land Resource, Chongqing Technology and Business University, Xuefu Avenue 19, Nan’an District, Chongqing, 400067 China; 3 Department of Soil and Environment, Institute of Mountain Hazards and Environment, Chinese Academy of Sciences, No. 9, Block 4, Renminnanlu Road, Chengdu, 610041 China

**Keywords:** DOM associate, Sorption competition, Sorption nonlinearity, Sorption strength, Spectroscopy, Treated wastewater

## Abstract

Pharmaceutically active compounds (PhACs) released into the environment have an adverse impact on the soil and water ecosystem as well as human health. Sorption of PhACs by soils and its potential modification through introduced DOM in the applied animal manure or treated wastewater (TWW) determines the mobility and environmental relevance of PhACs. Sulfadiazine, caffeine and atenolol were selected as target PhACs to investigate their sorption behaviors by five selected arable soils in the absence and presence of pig manure DOM. Sulfadiazine was least sorbed, followed by caffeine and atenolol according to the Freundlich sorption isotherm fit (soil average *K*_*f*_ [μg^(1−n)^ mL^n^ g^−1^] 4.07, 9.06, 18.92, respectively). The addition of manure DOM (31.34 mg C L^−1^) decreased the sorption of sulfadiazine and especially of caffeine and atenolol (average *K*_*f*_ 3.04, 6.17, 5.79, respectively). Freundlich sorption isotherms of the PhACs became more nonlinear in the presence of manure DOM (Freundlich exponent *n* changed from 0.74**–**1.40 to 0.62**–**1.12), implying more heterogeneous sorption of PhACs in soil**–**DOM binary systems. Sorption competition of DOM molecules with sulfadiazine and caffeine mostly contributed to their decreased soil sorption when DOM was present. In contrast, the formation of DOM**–**atenolol associates in the solution phase caused the largely decreased soil sorption of atenolol in the presence of DOM. It is suggested that DOM concentration (e.g., ≥ 60 mg C L^−1^) and its interaction with PhACs should be taken into consideration when assessing the environmental impact of land application of animal manure or irrigation with TWW.

**Supplementary Information:**

The online version contains supplementary material available at 10.1007/s10653-021-00904-3.

## Introduction

In recent decades, concerns are growing about the environmental effects of pharmaceutical active compounds (PhACs) due to their increasing consumption and frequent detection in different environmental matrices all over the world (aus der Beek, 2016). Inputs of PhACs from veterinary medicines via land application of animal manure and from human medicines via irrigation of treated wastewater (TWW) lead to the enrichment of PhACs in arable topsoils (Keerthanan et al., 2020). Soil exposure to PhACs can suppress soil microbial activities or decrease microbial biomasses, and also increase the occurrence of resistance genes in the ecosystem (McKinney et al., 2018; Wang, Wang, et al., 2018). In addition, root uptake of PhACs and their transformation products by edible plants and/or leaching of PhACs to the groundwater also increase the chronic exposure of humans to PhACs (Hammad et al., 2018; Spielmeyer et al., 2017). Sorption of the PhACs to soil is a major determinant regarding the fate and mobility of PhACs in subsurface systems (Wang & Wang, 2015).

PhACs’ structures and associated chemical properties (e.g., amphiphilicity, polarity) are highly related to their sorption as these characteristics influence their charge localization, surface complexation and affinities to soils (Carrasquillo et al., 2008; Vulava et al., 2016). The interplay between the physicochemical properties of PhACs and soils determines the sorption of PhACs. Soil pH largely influences the sorption of PhACs especially through its impact on the speciation (i.e., cationic, neutral, anionic or zwitterionic) of ionizable PhACs (Kodešová et al., 2015). The soil constituent most relevant for sorption is soil organic matter (SOM), which is mainly due to specific PhAC-SOM associations (Arye et al., 2011; Pavlović et al., 2018; Vulava et al., 2016), while Fe oxides and clay minerals also facilitate the sorption of PhACs especially in SOM-poor soil (Yamamoto et al., 2018).

Furthermore, PhACs, other than, e.g., pesticides, typically reach soils not in aqueous solution but in organic waste substrates such as manure and TWW. These waste substrates are characterized by high contents of dissolved organic matter (DOM) that interferes with the sorption of PhACs (Thiele-Bruhn & Aust, 2004; Zhou et al., 2016). Decreased sorption of certain PhACs (e.g., chlortetracycline, tylosin and sulfapyridine) to soils and enhanced transport (e.g., tetracycline, ibuprofen and sulfamethoxazole) in a soil due to the addition of manure-derived DOM have been reported (Chabauty et al., 2016; Haham et al., 2012; Lee et al., 2014). Manure amended and/or TWW irrigated soil can be a long-term source of groundwater contamination with PhACs, which is probably due to the mobilizing effect of DOM on PhACs (Blackwell et al., 2009; Spielmeyer et al., 2017). In contrast, increased sorption affinity of PhACs (sulfadiazine, ofloxacin and carbamazepine) to soils in the presence of exogenous DOM was reported elsewhere (Li et al., 2019; Navon et al., 2011; Sukul et al., 2008). Both mobilizing and retaining effects of manure DOM on the sorption of the same PhAC (sulfamethazine) to different soils were found and attributed to different sources and composition of manure DOM (Chu et al., 2013; Lee et al., 2014). These apparently contradicting results reveal the necessity to study in more detail the effect of manure DOM on the sorption of PhACs to dissimilar arable soils in comparison.

In this study, sulfadiazine, caffeine and atenolol were selected as typical and widely used veterinary and human PhACs to investigate their sorption to five arable topsoils with different properties in the presence and absence of manure DOM. Sulfadiazine is an extensively used veterinary antibiotic of the sulfonamides group that typically enters soil with animal manure (Łukaszewicz et al., 2017). Atenolol is a commonly used β-blocker drug for cardiovascular diseases treatment. Caffeine is a widely used psychoactive drug used as central nervous system stimulant and even more a daily consumable product from beverages such as coffee and tea (Rainsforth 2017). Both atenolol and especially caffeine are ubiquitous micropollutants in municipal wastewater, resulting in high emission into soil via TWW irrigation (Gros et al., 2010; Huerta-Fontela et al., 2008; Lindim et al., 2016). Both animal manure and TWW containing these PhACs are characterized by their high content of DOM that is supposed to influence the sorption of PhACs to soils. Therefore, major objectives of this study are (1) to reveal the sorption behavior of veterinary and human PhACs to arable topsoils of various characteristics, (2) to explore the interaction of manure DOM with soil and (3) to investigate the effects of manure DOM on the sorption of PhACs.

## Materials and methods

### Chemicals

Sulfadiazine, caffeine and atenolol of analytical grade were purchased from Dr. Ehrenstorfer GmbH (Augsburg, Germany). Molecular structures and selected properties of the target PhACs are presented in Table S1 (in Electronic Supplementary Material). Stock solutions of PhACs were prepared by dissolving 10 mg of each compound in 10 ml of methanol and were stored at 4 °C. Speciation of PhACs (shown in Fig. S1 a, b, c) was calculated using *pKa* values in Table S1 and Eqs. S1–S5.

### Soils and manure DOM

Pharmaceutical sorption experiments were conducted for five agricultural soils (Haplic and stagnic Cambisols). Topsoils (0–15 cm) were sampled from different arable fields in the greater region of Trier, Germany. The five soils sampled were recorded as soil I (N 49° 49, 67′; E  6° 25, 66′), II (N 49° 51, 77′; E 6° 49, 26′), III (N 49° 52, 02′; E 6° 23, 67′), IV (N 49° 52, 30′; E  6° 24, 95′) and V (N 49° 43, 03′; E 6° 42, 82′). They were selected to cover typical ranges of properties of agricultural soils in temperate regions, i.e., a) pH from 4.28 to 6.05; b) SOC content from 1% to 2.5%; c) soil texture from sandy to clayey soils. Soil samples were air-dried and sieved to < 2 mm. General properties of the soils are presented in Table 1. Soil pH and electrical conductivity (EC) were determined by a pH meter (electrode SenTix 21, WTW, Germany) and conductivity meter (Cond 340i, WTW, Germany), respectively, in 0.01 M CaCl_2_ with a soil to solution ratio of 1:2.5. Organic carbon (OC) content of soil and manure, respectively, was measured using an elemental analyzer (EA 3000, Hekatech, Wegberg, Germany). Oxalate extractable iron oxides (Fe_o_) were determined using the modified method from Schwertmann (1964) and by graphite furnace atomic absorption spectrometry (ContrAA 700 High Resolution Continuum Source, Varian, Palo Alto, CA).

**Table 1:** Selected physicochemical properties of the five tested topsoil samples

Soil	pH_CaCl2_	EC (mS cm^−1^)	OC (g kg^−1^)	Fe_o_ (%)	Clay (%)	CEC (mmol_c_ kg^−1^)	Texture class
I	5.48	2.63	16.77	0.10	4	56.40	Sandy loam
II	4.28	2.43	11.57	0.18	6	37.18	Loamy sand
III	5.59	2.58	18.10	0.34	38	93.45	Clay loam
IV	6.05	2.78	25.69	0.35	15	66.04	Silt loam
V	6.01	2.65	11.68	0.38	12	80.02	Silt loam

Liquid pig manure was used (i) because it is the manure type most relevant for veterinary PhACs import into soils and (ii) to also represent human sewage and TWW, since human and pig diet and digestion are largely similar. Pig manure was obtained from an agricultural farm in Niederfeulen, Luxemburg, rearing 11,500 animals. Manure storage was done within the stable under slatted floor. None of the PhACs investigated in this study was used on the farm, so that the manure was free from residues of the three PhACs.

Homogenized liquid manure was centrifuged (15,000 g, 45 min) before two subsequent filtrations of the supernatant through (i) a 1-μm GF/F glass fiber filter (Whatman, UK) and (ii) a 0.45-μm cellulose-acetate filter membrane (Sartorius, Germany), to receive manure DOM. The obtained manure DOM fraction was freeze-dried for further use in batch sorption experiments. The OC content of the freeze-dried manure DOM was 156.7 g kg^−1^.

### Batch sorption experiments

Two types of batch sorption experiments were carried out, i.e., the sorption of manure DOM alone to soils and the sorption of PhACs to soils with or without the presence of manure DOM. For the latter, both effects of manure DOM at a single concentration on PhACs’ sorption isotherms and of varied concentrations of manure DOM on the sorption of PhACs added at a single spiking concentration were explored. According to the preliminary tests, soil pH showed little variations in the presence of manure DOM, resulting in < 4% of variation of the speciation of sulfadiazine, caffeine and atenolol. Therefore, no pH control was conducted in the batch sorption experiments.

To investigate single point sorption of manure DOM, soil samples were suspended in 0.01 M CaCl_2_ solutions (soil:water = 1:2.5 w/w) and allowed to equilibrate for 24 h. Subsequently, freeze-dried manure DOM was added at a final concentration of 31.34 mg C L^−1^, which was comparable to typical DOM levels in soil (3–75 mg C L^−1^) (Oren & Chefetz, 2012). Respective samples without addition of manure DOM served as controls. All samples (in triplicates) were agitated on an end-over-end shaker at 22 °C at 15 rpm for 72 h (equilibrium time was determined in preliminary kinetic experiment) before centrifugation at 2000 g for 30 min. The supernatant was decanted and filtered through a 0.45-μm cellulose-acetate filter membrane (Sartorius, Germany). The filtrate was analyzed for DOC concentration using a TOC/N analyzer (TOC-Vcpn, Shimadzu, Duisburg, Germany). The soil-sorbed DOC was calculated as the difference between the introduced DOC from manure DOM plus soil-derived DOC in control samples and the remaining DOC in the filtrate. Chemical properties and possible sorption mechanisms of manure DOM were determined by spectroscopic analyses. The absorption of the filtered supernatant was measured with a UV/VIS spectrophotometer (Shimadzu, Japan) in the wavelength range from 200 to 800 nm. The specific UV absorbance at 280 nm (SUVA_280_), determined by normalizing the absorbance at 280 nm of each filtered supernatant by the respective TOC concentration, and the E_2_/E_3_ ratio, determined as the ratio between the absorbance at 250 nm and 365 nm of each sample, were calculated thereafter for the characterization of DOM (Yang et al., 2020). SUVA_280_ and E_2_/E_3_ serve as indices for sample aromaticity and molecular size, respectively. In addition, soils equilibrated with manure DOM, i.e., associates of soil with sorbed manure DOM, were collected following centrifugation and freeze-dried. The original soil samples, manure DOM (before soil contact) as well as soil–manure DOM associates were analyzed by FTIR (Nicolet 560, USA) in a wavenumber range from 400 to 4000 cm^−1^.

To determine PhACs sorption to soils, batch sorption experiments were carried out according to OECD guideline 312 (OECD, 2004). Five grams of each soil were suspended in 12.5 ml 0.01 M CaCl_2_ (soil:solution = 1:2.5 w/w) in polypropylene centrifuge tubes and shaken overnight. Afterward, three PhACs were spiked separately into the soil suspension with initial concentrations of PhACs in the range of 1–100 μg g^−1^. In order to investigate the effect of manure DOM on PhACs sorption, freeze-dried manure DOM was added at an amount corresponding to 31.34 mg C L^−1^ along with each PhAC to separate samples. All samples were prepared in triplicate plus two blanks without soil and PhACs. Sample agitation and centrifugation were kept the same as described above for single point sorption of manure DOM. Furthermore, the impact of six varied initial manure DOM concentrations in the range from 0 to 140 mg C L^−1^ on the sorption of PhACs (single spiking content 50 μg g^−1^) were carried out. Sample preparation and handling were kept the same as described before.

In addition, the occurrence of PhAC-manure DOM associates was determined. To this end, supernatants from the batch sorption experiments with PhAC spiking concentration of 100 μg g^−1^ and manure DOM were divided into equal aliquots. Aliquot (1) was filtered through 0.45-μm cellulose-acetate filter membrane before clean-up to receive both DOM and liquid phase. DOM was removed from aliquot (2) by adding CaCl_2_ at a final concentration of 0.5 M Ca^2+^. Coagulation of manure DOM is facilitated through binding of Ca^2+^ by non-specific electrostatic interactions and Ca^2+^-cation bridge formation (Li et al., 2020; Zheng et al., 2020). Subsequently, both aliquots were centrifuged and filtered as described before. Differences in the contents of the target PhACs in the two aliquots were attributed to the occurrence of PhAC-manure DOM associates in the solution phase.

### Sample clean-up and analysis of PhACs by LC–MS/MS

For PhAC analysis, the decanted supernatants were cleaned up by solid phase extraction (SPE) using a HR-X cartridge (Macherey–Nagel, Düren, Germany). The HR-X material was preconditioned with 6 ml of methanol followed by 6 ml of HPLC grade water. After the supernatant was passed through the cartridge, it was rinsed with 6 ml methanol:water mixture (2:8 v/v) and subsequently dried in a gas stream for 30 min. Finally, the target PhACs were eluted from the cartridge using 6 ml methanol. The eluted volume was evaporated to < 0.5 ml in a rotary evaporator (Rotavapor R-114, Switzerland) and redissolved in 1.0 ml methanol. Samples were spiked with 10 μL sulfadimidine (50 μg mL^−1^ in methanol) as internal standard for quantification, and transferred to amber LC autosampler vials. The average recoveries of sulfadiazine, caffeine and atenolol were 84.03%, 90.15% and 79.49%, respectively.

Chromatographic separation of the analytes was performed on a Hypersil Gold C18 HPLC column (50 × 2.1 mm, 3.0 µm, Thermo Electron, USA) using (eluent A) HPLC water with 0.1% formic acid (v/v) and (eluent B) methanol with 0.1% formic acid (v/v) as mobile phases. The gradient program was as follows: initial condition 98% of A, followed by a 10-min linear gradient to 100% of B, 4-min isocratic elution and 1-min linear gradient back to 98% of A, which was held for 2 min to equilibrate the column. The flow rate was maintained at 0.2 mL min^−1^ and the injection volume was 10 µL.

The chromatographic system used for analysis consisted of a Shimadzu LC-20 HPLC (Shimadzu, Duisburg, Germany) coupled to an API 3200 LC–ESI–MS/MS (Applied Biosystems/MDS Sciex Instruments, Toronto, Canada) operated in positive ion mode. The ion-source settings were: ion spray voltage 5000 V; source temperature 400 °C; curtain gas 25 psi; collision gas 7 psi. Peak integration and data evaluation of the measurements were performed with the Analyst 1.4.2 software (Applied Biosystems/MDS Sciex Instruments, USA). Evaluation of chromatograms was done as reported by Ngigi (2020). The limit of detection of the analytical method was 5 µg L^−1^and the limit of quantification (LOQ) was10 µg L^−1^. All data reported here were above the LOQ.

### Data analysis

Based on previous work (e.g., Kiecak et al., 2019; Thiele-Bruhn et al., 2004), the Freundlich, Langmuir and linear models were used for the fitting of sorption isotherms. Because the Freundlich model was superior to the other two models and the Langmuir equation failed to model several data sets, only results and sorption isotherms fitted with the Freundlich model are shown.

1 $$C_{s} = K_{f} C_{e}^{n} ,$$

where *K*_*f*_ (μg^(1−n)^ mL^n^ g^−1^) is the Freundlich unit sorption capacity and *n* is a measure of isotherm nonlinearity. The linear sorption coefficient (*K*_*d*_) describes the association favorability of the analyte to the sorbent and was calculated from *K*_*f*_ using the following equation (Navon et al., 2011).

2 $$K_{d} = K_{f} C_{e}^{n - 1} ,$$

*K*_*d*_ (mL g^−1^) was calculated at equilibrium concentrations (*C*_*e*_) of 10 mg L^−1^ in this study.

In addition, Pearson correlations were calculated to determine the relation between sorption coefficients (*K*_*f*_, *K*_*d*_) and sorbent properties. Due to the limited number of five soils and respective soil data, one-tailed correlation was used and significance was accepted at the level of *p* < 0.1.

Effects of different manure DOM spiking contents on PhACs’ sorption were fitted by nonlinear regression using the Curve Expert Professional 2.6.5 software (Hyams Development, USA). Based on the best-fit method, the linear–quadratic–rationale model was selected from a set of tested models (data not shown).

## Results and discussion

### Sorption of PhACs to soils

**Fig. 1:**
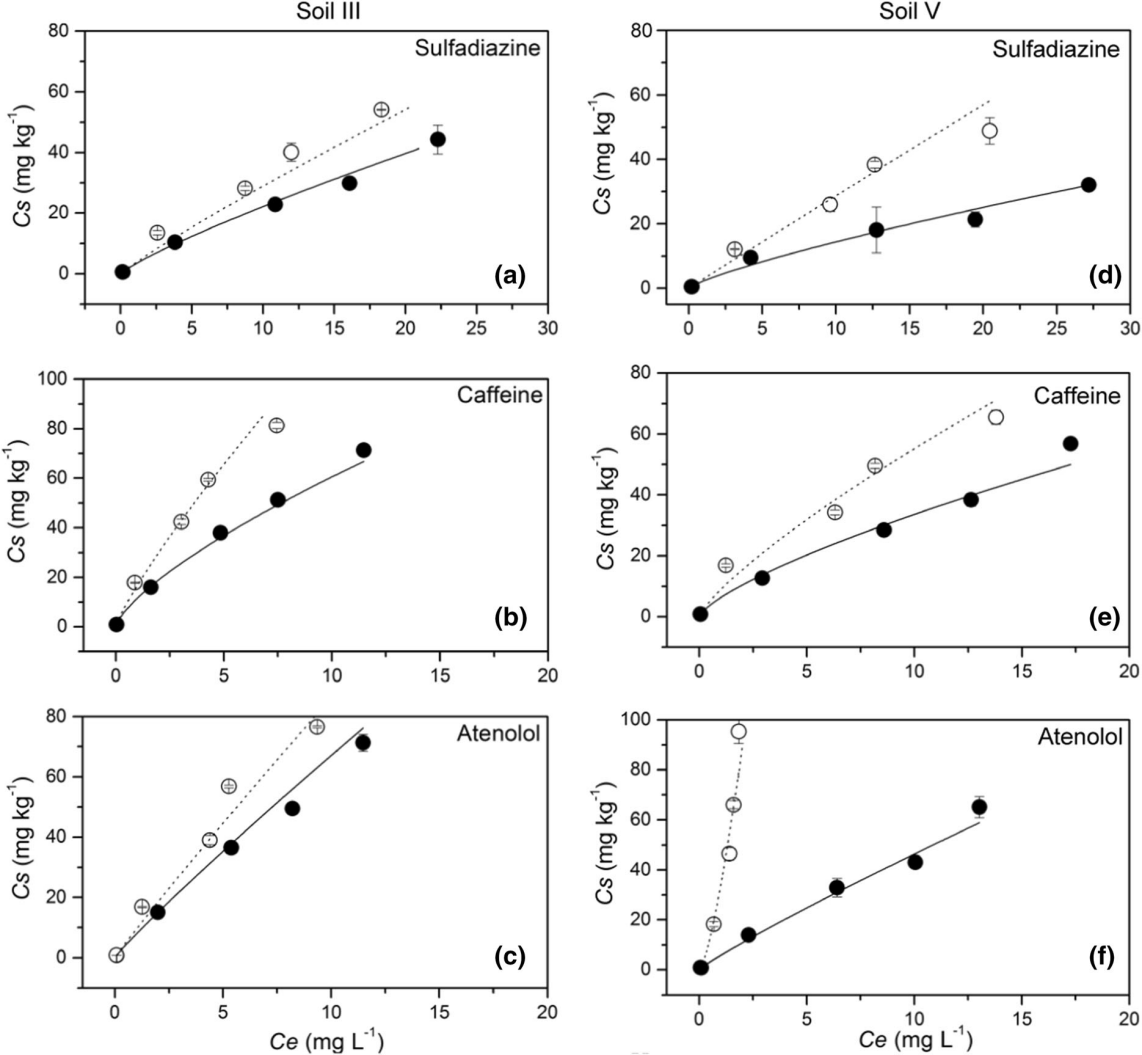
Freundlich sorption isotherms of sulfadiazine, caffeine and atenolol in soil III **a**–**c** and soil V **d**–**f** in the presence (filled circles) and absence (open circles) of manure DOM; lines are curve fits using the Freundlich equation; error bars indicate standard errors of three replicate samples (bars not shown are smaller than the filled symbols)

The curve fit using the Freundlich equation yielded very good results with coefficients of determination (R^2^) ≥ 0.96 and standard deviation (SD) of ≤ 0.23 (Table 2). Representative Freundlich sorption isotherms for the three PhACs are shown in Fig. 1 on examples of soil III and V. Isotherms of sorption to the other three soils are shown in Figs. S2–S4 and all data of the curve fit are listed in Table 2. Most isotherms were clearly nonlinear with the exponents of nonlinearity (*n*) ranging from 1 to 0.74 (Table 2). The *n* values of caffeine sorption were always the lowest of all three PhACs (0.74–0.88). Similar nonlinear sorption of caffeine to agricultural soils and comparable *n* values (0.67–0.81) were reported elsewhere (Zhang et al., 2013). In contrast, Freundlich exponents of atenolol sorption to soils ranged from *n* < 1 for soils III and IV with higher SOC content to *n* > 1 (up to *n* = 1.40) for soils I, II and V with low SOC content (Table 1). The differing *n* values of atenolol sorption are presumably related to different mechanisms of sorption to the five different soils; *n* values < 1 indicate site-specific chemi- and physisorption (Maszkowska et al., 2014; Thiele-Bruhn et al., 2004) while *n* values > 1 indicate multilayer sorption that was previously described for atenolol (Le Guet et al., 2018).

**Table 2:** Freundlich model parameters (*K*_*f*_ and *n*) as well as derived linear sorption coefficient (*K*_*d*_) for soil sorption of PhACs in the presence and absence of manure DOM (mDOM); standard deviation of fitted parameters and whole model are in parentheses

Sample	Sulfadiazine				Caffeine				Atenolol			
	K_f_	n	R^2^	K_d_	K_f_	n	R^2^	K_d_	K_f_	n	R^2^	K_d_
I	3.12	0.96	0.99	2.83	3.84	0.74	0.97	2.10	36.91	1.10	1.00	46.04
	(0.13)	(0.05)	(0.23)	(0.25)	(0.16)	(0.01)	(0.14)	(0.04)	(2.00)	(0.01)	(0.05)	(3.45)
I + mDOM	2.90	0.94	1.00	2.50	3.39	0.71	1.00	1.75	6.74	1.00	0.99	7.46
	(0.33)	(0.01)	(0.06)	(0.28)	(0.13)	(0.01)	(0.05)	(0.01)	(0.06)	(0.01)	(0.11)	(0.08)
II	3.08	0.90	0.99	2.44	3.99	0.74	0.99	2.20	4.94	1.24	0.96	8.58
	(0.01)	(0.01)	(0.09)	(0.08)	(0.21)	(0.03)	(0.09)	(0.07)	(0.10)	(0.04)	(0.20)	(0.80)
II + mDOM	2.09	0.82	0.99	1.39	2.53	0.62	0.99	1.06	2.90	1.12	1.00	3.82
	(0.01)	(0.03)	(0.08)	(0.10)	(0.14)	(0.03)	(0.06)	(0.03)	(0.30)	(0.08)	(0.05)	(0.82)
III	4.07	0.93	0.99	3.45	16.94	0.87	1.00	12.41	10.15	0.93	0.99	8.56
	(0.04)	(0.02)	(0.11)	(0.15)	(0.40)	(0.01)	(0.06)	(0.04)	(0.60)	(0.01)	(0.10)	(0.32)
III + mDOM	3.21	0.82	0.99	2.11	11.64	0.72	1.00	6.05	7.73	0.91	1.00	6.27
	(0.08)	(0.02)	(0.05)	(0.14)	(0.23)	(0.02)	(0.03)	(0.17)	(0.11)	(0.01)	(0.07)	(0.01)
IV	5.77	0.92	1.00	4.77	11.59	0.88	0.96	8.79	9.01	0.97	1.00	8.43
	(0.30)	(0.01)	(0.09)	(0.09)	(0.53)	(0.04)	(0.18)	(1.22)	(0.29)	(0.02)	(0.06)	(0.56)
IV +	5.09	0.87	1.00	3.75	7.01	0.72	0.99	3.65	5.78	0.97	1.00	5.34
mDOM	(0.15)	(0.01)	(0.03)	(0.19)	(0.50)	(0.03)	(0.05)	(0.02)	(0.91)	(0.01)	(0.05)	(0.95)
V	4.30	0.87	0.99	3.19	8.96	0.79	0.98	5.51	33.61	1.40	1.00	84.61
	(0.87)	(0.22)	(0.15)	(0.45)	(0.28)	(0.01)	(0.12)	(0.41)	(3.64)	(0.06)	(0.06)	(1.91)
V + mDOM	1.91	0.85	0.99	1.36	6.30	0.73	1.00	3.35	5.79	0.90	1.00	4.62
	(0.43)	(0.01)	(0.08)	(0.22)	(0.46)	(0.01)	(0.04)	(0.14)	(0.24)	(0.01)	(0.04)	(0.31)

Among the three PhACs tested, sulfadiazine always exhibited the least sorption affinity to all five soils with average values for *K*_*f*_ (μg^(1−n)^ mL^n^ g^−1^) and *K*_*d*_ (mL g^−1^) of 4.07 and 3.34, as compared to 9.06 and 6.15 for caffeine as well as 18.92 and 31.24 for atenolol. Comparable low *K*_*f*_ values of sulfadiazine sorption to soils were also reported in other studies (e.g., Conde-Cid et al., 2019; Shen et al., 2018; Thiele-Bruhn et al., 2004). All three PhACs have about similarly low *K*_*OW*_ values of 0.812, 0.851 and 1.445 for sulfadiazine, caffeine and atenolol, respectively (Table S1), reflecting a low potential for soil sorption through hydrophobic partitioning. Additionally, *K*_*OW*_ values do not match with the determined data for *K*_*f*_ and *K*_*d*_. This finding is in accordance with other reports (Gros et al., 2019; Kiecak et al., 2019) and suggests that other mechanisms were more important in controlling the sorption of the three PhACs to soils. The relatively much lower sorption of sulfadiazine was attributed to its speciation. Sulfadiazine occurred as anionic (mass fraction 0.60–26.2%) and neutral species (mass fraction 73.8–99.2%) depending on soil pH (Fig. S1a). Both species but especially the anionic species are unfavorable for sorption to the negatively charged soil surfaces. In contrast, the cationic forms (mass fraction > 99.7%, Fig. S1b, c) of caffeine and atenolol present in the five soils facilitate electrostatic bonding to the negatively charged soil surfaces, resulting in higher sorption affinities of these two PhACs (Xu et al., 2021). The results of this study are consistent with other findings, showing a significantly higher sorption (around 10–20-fold) of cationic PhACs such as caffeine and atenolol, compared to PhACs with non-cationic species (Kodešová et al., 2015; Miroslav et al., 2018).

As expected, the sorption strengths of the three PhACs varied with the different properties of the five tested soils. The *K*_*f*_ values were correlated with some of the basic soil properties (Table 3). These were for sulfadiazine the Fe_o_ and SOC content (*R* > 0.75; *p* < 0.1) and for caffeine the clay content (*R* = 0.95; *p* < 0.01) of the five soils, confirming the reports on the predominant relevance of these soil components for the sorption of sulfadiazine and caffeine, respectively (Kiecak et al., 2019; Sukul et al., 2008). Moreover, CEC was significantly correlated to the sorption coefficients of caffeine (*R* = 0.86; *p* < 0.05), pointing out that cation-exchange is a controlling mechanism for the sorption of caffeine (Xu et al., 2021). In contrast, sorption coefficients of atenolol showed no correlation to any of the basic soil parameters, which contrasts previous findings where a significant, positive correlation of atenolol sorption with CEC, OC and clay content were reported (Kodešová et al., 2015; Yamamoto et al., 2018). Electrochemical interaction, ion exchange and/or charge transfer have been suggested as the potential mechanisms of atenolol sorption to various sorbents (Al-Khazrajy et al., 2016; Rakić et al., 2013; Schaffer et al., 2012), pointing to the fact that multimodal combinations of soil properties are relevant for atenolol sorption.

**Table 3:** Pearson correlation coefficients (one-tailed) of soil properties (n = 5) with *K*_*f*_ values of sulfadiazine (SDZ), caffeine (CAF) and atenolol (ATN) soil sorption with or without addition of manure DOM (mDOM)

	pH_CaCl2_	SOC	CEC	Fe_o_	Clay
*K*_*f*_					
SDZ	0.723^+^	0.752^+^	0.431	0.763^+^	0.312
CAF	0.528	0.469	0.863*	0.771^+^	0.949**
ATN	0.415	− 0.292	0.149	− 0.215	− 0.387
SDZ + mDOM	0.475	0.994**	0.161	0.258	0.252
CAF + mDOM	0.535	0.397	0.913*	0.716^+^	0.966**
ATN + mDOM	0.710^+^	0.419	0.817*	0.263	0.673

Significance at the 0.1, 0.05 and 0.01 level are indicated by symbols +, *, **, respectively

### Sorption of manure DOM to soils

Soil-derived DOM, determined as DOC contents in equilibrium solutions of soils without addition of manure DOM, ranged from 15.3 to 63.2 mg L^–1^across the five tested soils (Table 4). Added manure DOM was strongly retained by soil within 24 h of contact. This resulted in a much smaller increase in the DOC concentration (between 2.11 and 10.89 mg L^–1^) than expected from the addition of 31.34 mg manure DOC L^–1^ (Table 4). The sorption coefficient *K*_*d*_ of manure DOM to the five tested soils ranged between 4.69 and 34.56 mL g^–1^, which range overlapped with the *K*_*d*_ of animal waste DOM (0.78–20.25 mL g^–1^) in other soils (Huang & Lee, 2001). In part, higher sorption coefficients could be assigned to differences in the properties of the soils tested in this study and the study of Huang and Lee (2001). The *K*_*d*_ values of manure DOM sorption were significantly correlated with the clay contents of soils (*R* = 0.76; *p* < 0.1), confirming the relevant contribution of clay for soil sorption of DOM (Gmach et al., 2020; Oren & Chefetz, 2012). Furthermore, highest values of the *K*_*d*_ were found in soil III and IV with both highest content of clay and SOC. Generally, *K*_*d*_ of manure DOM was higher than that of the three PhACs. Therefore, competition for sorption sites on soil surfaces was expected for mixtures of PhACs and manure DOM.

**Table 4:** Coefficient (*K*_*d*_) of manure DOM (mDOM) sorption to soil as well as DOC content and UV–VIS parameters SUVA_280_ and E_2_/E_3_ of soil equilibrium solutions without or with addition of manure DOM (mDOM) at a spiking concentration of 31.34 mg DOC L^−1^

Sample	*K*_*d*_ (mL g^−1^)	DOC (mg L^−1^)	SUVA_280_ (L × mm g^−1^)	E_2_/E_3_
mDOM	−	−	2.22 ± 0.86	3.53 ± 0.21
I		63.21 ± 2.50	0.95 ± 0.07	7.40 ± 0.20
I + mDOM	4.69 ± 0.98	74.10 ± 1.86	1.00 ± 0.07	7.16 ± 0.32
II		38.45 ± 1.84	0.75 ± 0.01	6.14 ± 0.68
II + mDOM	17.77 ± 3.79	42.32 ± 2.14	0.95 ± 0.05	4.83 ± 0.23
III		38.31 ± 2.17	0.95 ± 0.20	5.27 ± 0.23
III + mDOM	34.56 ± 3.24	40.42 ± 1.23	1.20 ± 0.08	5.18 ± 0.01
IV		42.86 ± 3.28	0.98 ± 0.04	6.23 ± 0.21
IV + mDOM	33.29 ± 4.31	45.00 ± 1.11	1.08 ± 0.03	5.63 ± 0.19
V		15.31 ± 0.76	1.22 ± 0.01	6.26 ± 0.35
V + mDOM	25.27 ± 2.38	18.13 ± 2.14	1.72 ± 0.14	6.02 ± 0.10

Going along with the soil sorption of manure DOM, the chemical composition of solutions was strongly altered. Substantially lower values of SUVA_280_ but higher *E*_2_/*E*_3_ ratios of the solution from soil–manure DOM mixtures were observed in comparison with single manure DOM solution. This indicated that the aromaticity and molecular size of DOM in mixtures of soil with manure DOM was reduced compared to manure DOM alone and resembled much more the spectroscopic properties of soil-derived DOM without manure DOM addition. Consequently, a preferential binding of larger-sized or highly aromatic components of DOM to the soil matrix (clay minerals and also soil organic matter) might have occurred as it was previously reported (Engel & Chefetz, 2015, Oren & Chefetz, 2012; Sowers et al., 2019).

**Fig. 2:**
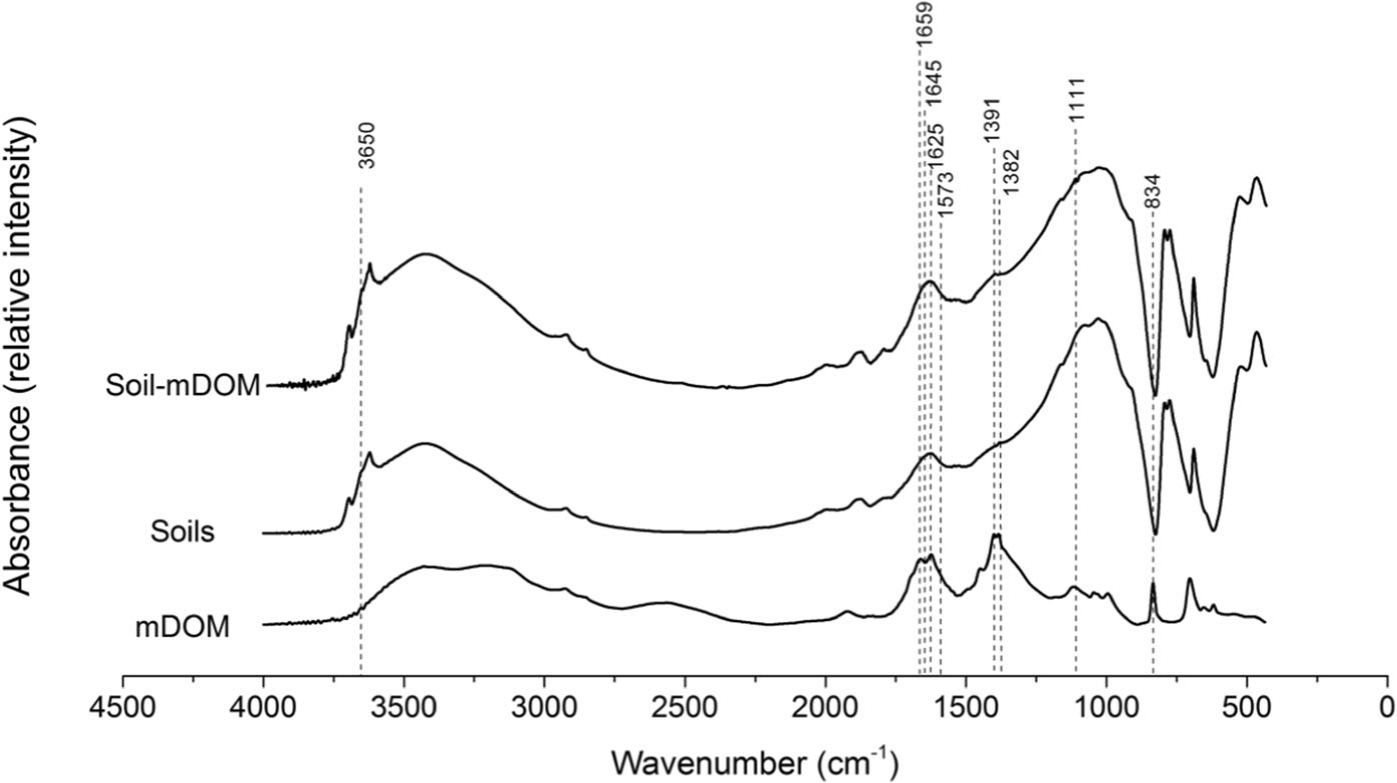
FTIR spectra of manure DOM (mDOM), soils and soil–mDOM associates. Spectra of soils and soil–mDOM mixtures shown here were averaged for all five soils

Analyzing freeze-dried manure DOM samples by FTIR revealed that the spectrum (Fig. 2) was characterized by significant contributions from aromatic CH groups (830 cm^−1^, 834 cm^−1^ in the current study), phenol groups (1111 cm^−1^, 3650 cm^−1^), carboxylic functional groups (1390 ± 10 cm^−1^), C = C vibration of aromatic structures (1625 cm^−1^) and N–C=O functional groups (1659 cm^−1^). The abundance of amide structures, phenolic, carboxylic and aromatic groups of manure DOM suggested a high level of maturity of the manure DOM (Huang & Lee, 2001) used in the current study. Although manure DOM was strongly sorbed to soil, it contributed only a small portion of at most 0.3 to 0.7% to the total OC in the soil samples. Hence, only small effects on spectroscopic properties of soil solids were expected from soil-sorbed manure DOM. Despite some small but visible features, the FTIR spectra were overall similar for all five soils. Therefore, averaged spectra of the five soils without and with addition of manure DOM are shown (Fig. 2). A local peak at 1111 cm^−1^ of the soil spectrum occurred after interaction with manure DOM. It corresponded to a small peak found in manure DOM that was not observed in the spectra of unamended soils. This result was ascribed to the interaction of the phenolic structures of manure DOM with soil minerals (Gu et al., 1995). The shoulder at 1382 cm^−1^ in the averaged soil spectrum shifted to 1391 cm^−1^ following the sorption of manure DOM (with a plateau between 1380 and 1400 cm^−1^), which was attributed to the complexation of carboxylate groups of manure DOM with metal oxides of the soils (Eusterhues et al., 2011; Kaiser & Guggenberger, 2007). A peak of manure DOM band at 1625 cm^−1^, accompanied by a shoulder at 1573 cm^−1^, was assigned to the aromatic C=C vibrations (Baes & Bloom, 1989; Biber & Stumm, 1994). A similar shoulder at 1625 cm^−1^ in the spectra of soils that had reacted with manure DOM indicated that the aromatic ring C=C structure of manure DOM was involved in the sorption to soils. The shoulder between 1645 and 1659 cm^−1^ in the soil spectrum became more pronounced when sorbed with manure DOM, which might be attributed to the interactions of C=O stretching vibrations in amides, aromatic C=C, COO– or hydrogen-bonded C=O groups of manure DOM with soils (Inbar et al., 1990; Keri et al., 2012). Zooming into the FTIR spectra, a small shoulder becomes visible between 3640 and 3650 cm^−1^ in the soil spectrum (position indicated by a dashed line in Fig. 2). This shoulder was changed to a small local peak at 3650 cm^−1^ after interaction with manure DOM, which corresponded to a small local peak determined in manure DOM at 3650 cm^−1^. According to the literature, a peak at around that wavenumber can be assigned to organic OH stretching (Chia et al., 2012). This further demonstrated the involvement of phenolic moieties of manure DOM molecules in the sorption to the soils.

### Effect of manure DOM addition on the sorption of PhACs

#### Sorption isotherms and parameters

Sorption of sulfadiazine, caffeine and atenolol in all five soils was lowered in the presence of manure DOM (Fig. 1; Figs. S2–S4; Table 2). The significant reduction of *K*_*f*_ values by a mean factor of 0.64 (0.17–0.93) went along with the reduction of the Freundlich exponent *n* by a mean factor of 0.90 (0.64–1.00). Similar mobilizing effects of DOM in soil and aquatic systems on sorbed pharmaceuticals such as sulfonamides (Chu et al., 2013; Haham et al., 2012; Thiele-Bruhn & Aust, 2004), caffeine (Nam et al., 2014) and atenolol (Le Guet et al., 2018) were reported.

The increased sorption nonlinearity (decreased *n*) of all three PhACs in the presence of manure DOM, suggested increased sorption heterogeneity when manure DOM was present. The effect of manure DOM on sorption strength and nonlinearity was notably strong for PhAC-soil combinations showing highest sorption coefficients (e.g., atenolol and soil I and V, caffeine and soil II and IV; Table 2). Such a different mobilization through DOM is a well-known effect for hydrophobic chemicals (Raber & Kögel-Knabner, 1997) and were here confirmed for polar chemicals as well.

Overall, rather similar correlations were found between soil properties and *K*_*f*_ values of PhAC sorption to soil in the presence of manure DOM (Table 3) for sulfadiazine and caffeine as were presented above for soils without manure DOM. Hence, the general sorption mechanisms of these two PhACs to the soil solid phase were not changed through manure DOM. Rather it contributed to a non-specific mobilization from the sorption sites as was previously reported for caffeine (Richards et al., 2017). In contrast, large variation of atenolol sorption relationship with soil properties occurred, i.e., *K*_*f*_ of atenolol sorption became significantly correlated to CEC (*p* < 0.05) in the presence of manure DOM (Table 3). This indicates that atenolol bound to soil through ion exchange preferentially resisted mobilization by DOM. It can be assumed that atenolol (–NH and –OH groups) interacted with manure DOM via charge transfer (Rakić et al., 2013), thus leading to sorption competition at respective soil sorption sites. The decreased sorption (*K*_*f*_) of the three PhACs in the presence of manure DOM was attributed to (i) sorption competition of manure DOM molecules at soil surfaces (see section on sorption of manure DOM to soils) and (ii) to the formation of PhAC-manure DOM associates that remain in solution. The latter was corroborated by the following experimental results.

Exclusion of DOM from PhAC analysis in equilibrium solutions yielded substantially lower concentrations of PhACs and the difference was assigned to PhAC-manure DOM associates (Table 5). The formation of such associates was especially strong for atenolol and declined in the sequence (average in parentheses): atenolol (50.7%) > caffeine (11.7%) ≥ sulfadiazine (9.4%). The larger affinity of atenolol to manure DOM reduces the sorption of atenolol to all five soils through co-mobilization by association with colloidal DOM. The percentage of PhAC-manure DOM associates and the difference in *K*_*d*_ (Δ*K*_*d*_; Table 5) were strongly and significantly correlated (*R* =  − 0.62; *p* < 0.05). However, this correlation was largely dominated by the data from atenolol. Sorption of PhACs to manure DOM possibly occurs via *π*–*π* interactions (Navon et al., 2011). Accordingly, this would be more relevant for the slightly less polar and less water-soluble atenolol (Table S1). In contrast, much lower percentages of PhAC-manure DOM associates were determined for sulfadiazine and caffeine. Considering the substantial decrease in soil sorption of both PhACs (indicated by *K*_*d*_ variation) in the presence of manure DOM, a strong competition of sulfadiazine and caffeine with manure DOM for the sorption sites on solid soil surfaces must be assumed, resulting in higher truly dissolved concentration of sulfadiazine and caffeine in the soil solution.

**Table 5:** Concentration of PhACs in equilibrium solution of soil with manure DOM (mDOM) addition (Aliquot 1) and after removal of DOM from solution (Aliquot 2), resulting percentage of PhAC-mDOM associates and DOM effect on the soil sorption coefficient *K*_*d*_

Soil	PhAC	PhAC (μg L^−1^)		PhAC-mDOM associates (%)^a^	Δ*K*_*d*_^b^
		Aliquot 1	Aliquot 2		(%)
I	SDZ	334.5 ± 7.1	269.0 ± 0.4	19.6	− 11.66
	CAF	151.3 ± 1.4	133.5 ± 2.1	11.8	− 16.67
	ATN	3.1 ± 0.1	1.6 ± 0.1	48.4	− 83.80
II	SDZ	331.8 ± 6.0	314.5 ± 10.6	5.2	− 43.03
	CAF	254.3 ± 0.4	251.5 ± 1.4	1.1	− 51.82
	ATN	106.5 ± 5.7	60.5 ± 0.71	43.2	− 55.48
III	SDZ	223.8 ± 1.1	205.5 ± 4.2	8.2	− 38.84
	CAF	88.5 ± 2.1	67.8 ± 0.4	23.4	− 50.16
	ATN	7.1 ± 0.2	2.8 ± 0.05	60.6	− 26.75
IV	SDZ	232 ± 5.7	216 ± 3.2	6.9	− 21.38
	CAF	66.3 ± 0.4	62.0 ± 1.4	6.5	− 58.48
	ATN	6.1 ± 0.7	5.2 ± 0.2	14.8	− 36.65
V	SDZ	301.8 ± 0.4	281.0 ± 1.4	6.9	− 57.37
	CAF	58.0 ± 0.1	48.9 ± 0.8	15.7	− 39.20
	ATN	30.8 ± 1.2	4.1 ± 0.2	86.7	− 94.54

^a^Percentage of the total concentration determined in aliquot 1

^b^Difference in *K*_*d*_ values of PhACs in the presence minus in the absence of manure DOM; calculated from data in Table S2. The initial spiking concentration of PhACs was 100 μg g^−1^

#### Influence of the spiking concentration of manure DOM

**Fig. 3:**
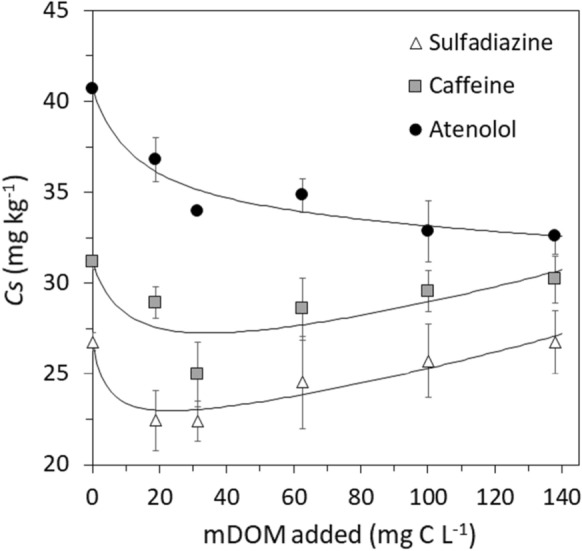
Effect of the initial manure DOM (mDOM) spiking concentration on the sorbed amount (*Cs*) of sulfadiazine (triangles), caffeine (squares) and atenolol (circles) to the five tested soils (averaged data). The spiking level of the three PhACs was in all samples at 50 μg g^−1^. Error bars not shown are smaller than symbols. Lines represent curve fits using the rational model

The DOM content of pig manure may vary with respect to type, age, husbandry conditions and diet of livestock animals as well as the technique of manure storage and management (Zhou et al., 2016). Different sources of wastewater will vary in DOM content as well (Graber & Gerstl, 2011). Hence, soil sorption of PhACs was further investigated under various initial manure DOM concentrations (0**–**140 mg C L^−1^). The effect of different manure DOM spiking concentrations on the sorbed amount of PhACs (*Cs*; mg kg^−1^) varied between soils but exhibited general trends that are shown by the results averaged for all five soils (Fig. 3). The sorption of atenolol consistently declined with increasing concentration of manure DOM, yet, the mobilizing effect declined as is shown by the nonlinear relationship. This corresponds to the assigned interaction mechanism of atenolol and manure DOM, forming atenolol**–**manure DOM associates that stayed in solution. Similarly, a decrease in oxytetracycline sorption to sediments goes along with an increase in added chicken manure DOM up to concentrations of 100 mg C L^−1^, while sorption is not further affected by higher added concentrations (Wang, Jiang, et al., 2018).

In contrast, the effect of increasing manure DOM on sulfadiazine and caffeine was non-monotonic. A mobilizing effect occurred up to a spiking concentration of 31.34 mg C L^−1^, while further increased concentrations had the opposite effect, leading to similar *Cs* at 0 and 140 mg C L^−1^ of added manure DOM. In agreement with the previously discussed interaction of sulfadiazine and caffeine with manure DOM at the soil sorption sites, it is speculated that higher spiking concentrations of manure DOM resulted in higher sorbed amounts of manure DOM as it was reported by Oren and Chefetz (2012). Based on spectroscopic parameters such as SUVA_280_ and E_2_/E_3_, it was demonstrated that rather aromatic components of manure DOM were preferentially sorbed to soil, forming new sorption sites. The non-monotonic curve shape in Fig. 3 indicates that with increasing DOM concentration and soil surface coverage, respectively, the sorption competition of manure DOM vanished in favor of the provision of new sorption sites for sulfadiazine and caffeine at the soil solid phase. Hence, it depends on the manure DOM content whether it has a mobilizing or immobilizing effect on certain PhACs. Such preferentially soil**-**sorbed aromatic and/or nonpolar DOM components originating from organic waste have also been demonstrated to facilitate sorption of other compounds, e.g., carbamazepine and estrogen (Navon et al., 2011; Stumpe & Marschner, 2010).

## Conclusions

Common agricultural practices such as land application of animal manure and irrigation with TWW, tend to spread contained PhACs as micropollutants onto the soil system, together with high amounts of DOM that are contained in these wastes. Batch sorption experiments are suitable to elucidate effects of manure DOM on soil sorption of relevant PhACs, i.e., sulfadiazine, caffeine and atenolol. Sorption isotherms of the PhACs to five arable soils were nonlinear with sorption coefficients declining in the sequence of atenolol > caffeine > sulfadiazine. The mobility of the three target PhACs was even aggravated in the presence of manure DOM (31.34 mg C L^−1^), which has significant implications for their environmental fate and relevance. The mechanisms of the mobilizing effect of manure DOM on the sorption of PhACs by soils differed among compounds. Competition of manure DOM molecules with sulfadiazine and caffeine for the sorption sites on soil surfaces likely caused the decreased soil sorption of these two PhACs. The preferential sorption of atenolol by manure DOM in solution led to a decreased soil sorption of atenolol. Additionally, the mobilizing effect of DOM on the sorption of PhACs was found to be related to the initial concentration of DOM among different PhACs. Increasing concentration of manure DOM up to 140 mg C L^−1^ led to consistent mobilizing of atenolol, while immobilization of sulfadiazine and caffeine occurred at a manure DOM spiking concentration higher than 31.34 mg C L^−1^. Considering such concentration-dependent mobilizing or immobilizing effect may help to explain the apparently contradicting findings in previous reports. It is expected that similar effects will occur with other substrates that are rich in DOM such as TWW. With respect to the adverse environmental effects of pharmaceuticals, the DOM content of various substrates including animal manure and TWW as well as their interaction with target PhACs in soils should be taken into consideration. Further research is needed regarding the impact of different chemical compositions of waste material-derived DOM.

## Supplementary Information

Below is the link to the electronic supplementary material.

Supplementary file1 (DOCX 915 KB)

## Data Availability

Additional data are available in the Supporting information.
