# Supplementary material for: Interaction of pig manure-derived dissolved organic matter with soil affects sorption of sulfadiazine, caffeine and atenolol pharmaceuticals
Source: Environ Geochem Health. 2021 Apr 15;43(10):4299–313. doi: 10.1007/s10653-021-00904-3 (PMC8473328; doi:10.1007/s10653-021-00904-3)
Supplement: Supplementary file 1 — Supplementary file1 (DOCX 915 KB) [file 10653_2021_904_MOESM1_ESM.docx]

**Interaction of pig manure derived dissolved organic matter with soil affects sorption of sulfadiazine, caffeine and atenolol pharmaceuticals**

Supporting information

# Wei Zhang • Xiangyu Tang • Sören Thiele-Bruhn

# W. Zhang

# Present address: School of Tourism and Land Resource, Chongqing Technology and Business University

# Xuefu Avenue 19, Nan'an District, Chongqing 400067, China

# W. Zhang • S. Thiele-Bruhn (🖂)

# Soil Science, University of Trier

# Behringstraße 21, 54296 Trier, Germany

# e-mail: thiele@uni-trier.de

# X. Tang

# Dept. of Soil and Environment, Institute of Mountain Hazards and Environment, Chinese Academy of Sciences

# No. 9, Block 4, Renminnanlu Road, Chengdu 610041, China

Equations for the calculation of sulfadiazine speciation at different pH:

$$\begin{matrix} \alpha_{cationic}(\%)=\frac{100}{(1+10^{(pH-pKa1)}+10^{(2pH-pKa1-pKa2)})} (Eq. S1) \\ \alpha_{neutral}(\%)=\frac{100}{(1+10^{(pKa1-pH)}+10^{(pH-pKa2)}) (Eq. S2)} \\ \alpha_{anionic}(\%)=\frac{100}{(1+10^{(pKa2-pH)}+10^{(pKa1+pKa2-2pH)})} (Eq. S3) \end{matrix}$$

Equations for the calculation of caffeine and atenolol speciation at different pH:

$$\begin{matrix} \alpha_{cationic}(\%)=\frac{100}{(1+10^{(pH-pKa)})} (Eq. S4) \\ \alpha_{neutral}(\%)=\frac{100}{(1+10^{(pKa-pH)})} (Eq. S5) \end{matrix}$$

**Table S1** Molecular structure and chemical properties of sulfadiazine, caffeine and atenolol

| Compound | Molecular structure | CAS number | Molar mass  (g mol^−1^) | p*K*a | | *K_ow_* | Water solubil-ity  (mg L^−1^) |
| --- | --- | --- | --- | --- | --- | --- | --- |
|  |  |  |  | 1 | 2 |  |  |
| Sulfadiazine | 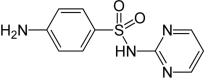 | 68-35-9 | 250.3 | 1.57^a^ | 6.50^a^ | 0.812^a^ | 2000 |
| Caffeine | 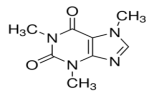 | 58-08-2 | 194.19 | 10.4^b^ |  | 0.851^c^ | 21600 |
| Atenolol | 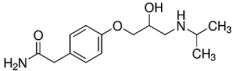 | 29122-68-7 | 266.34 | 9.6^b^ |  | 1.445^b^ | 429 |

a: Sukul et al. (2008).

b: <http://www.drugbank.ca>.

c: http://www.toxnet.nlm.nih.gov

**Table S2** *K_d_* values for three PhACs in the five soils without or with the addition of manure DOM (mDOM); calculated at equilibrium concentration (*Ce*) of 10 mg L^−1^

| Treatment | *K_d_* (mL g^−1^) | | |
| --- | --- | --- | --- |
|  | Sulfadiazine | Caffeine | Atenolol |
| Ⅰ | 2.83 | 2.1 | 46.04 |
| Ⅰ + mDOM | 2.5 | 1.75 | 7.46 |
| Ⅱ | 2.44 | 2.2 | 8.58 |
| Ⅱ + mDOM | 1.39 | 1.06 | 3.82 |
| Ⅲ | 3.45 | 12.14 | 8.56 |
| Ⅲ + mDOM | 2.11 | 6.05 | 6.27 |
| Ⅳ | 4.77 | 8.79 | 8.43 |
| Ⅳ + mDOM | 3.75 | 3.65 | 5.34 |
| Ⅴ | 3.19 | 5.51 | 84.61 |
| Ⅴ + mDOM | 1.36 | 3.35 | 4.62 |

**
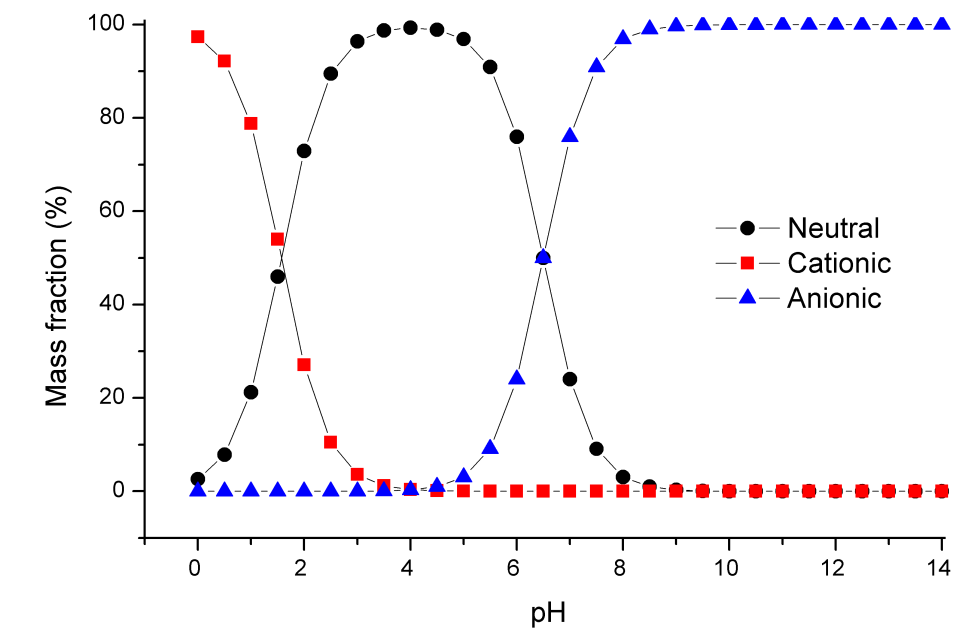
(a)**


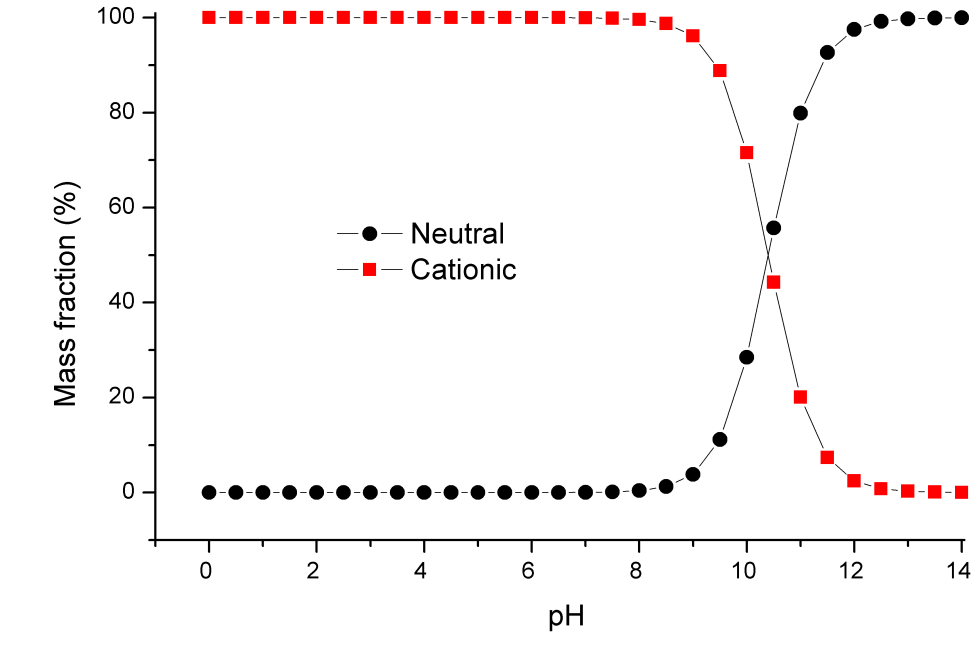
**(b)**

**(c)**
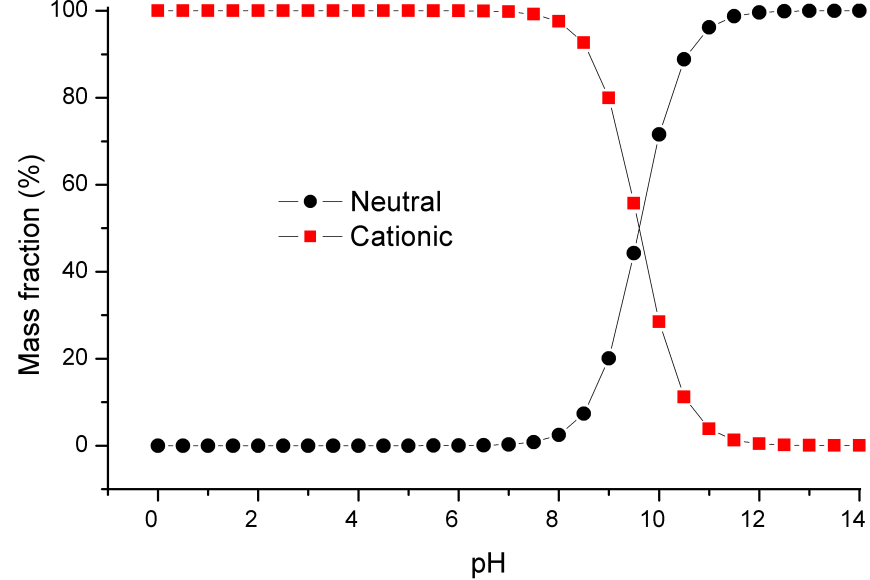


**Fig. S1** Speciation of (a) sulfadiazine, (b) caffeine and (c) atenolol at different pH


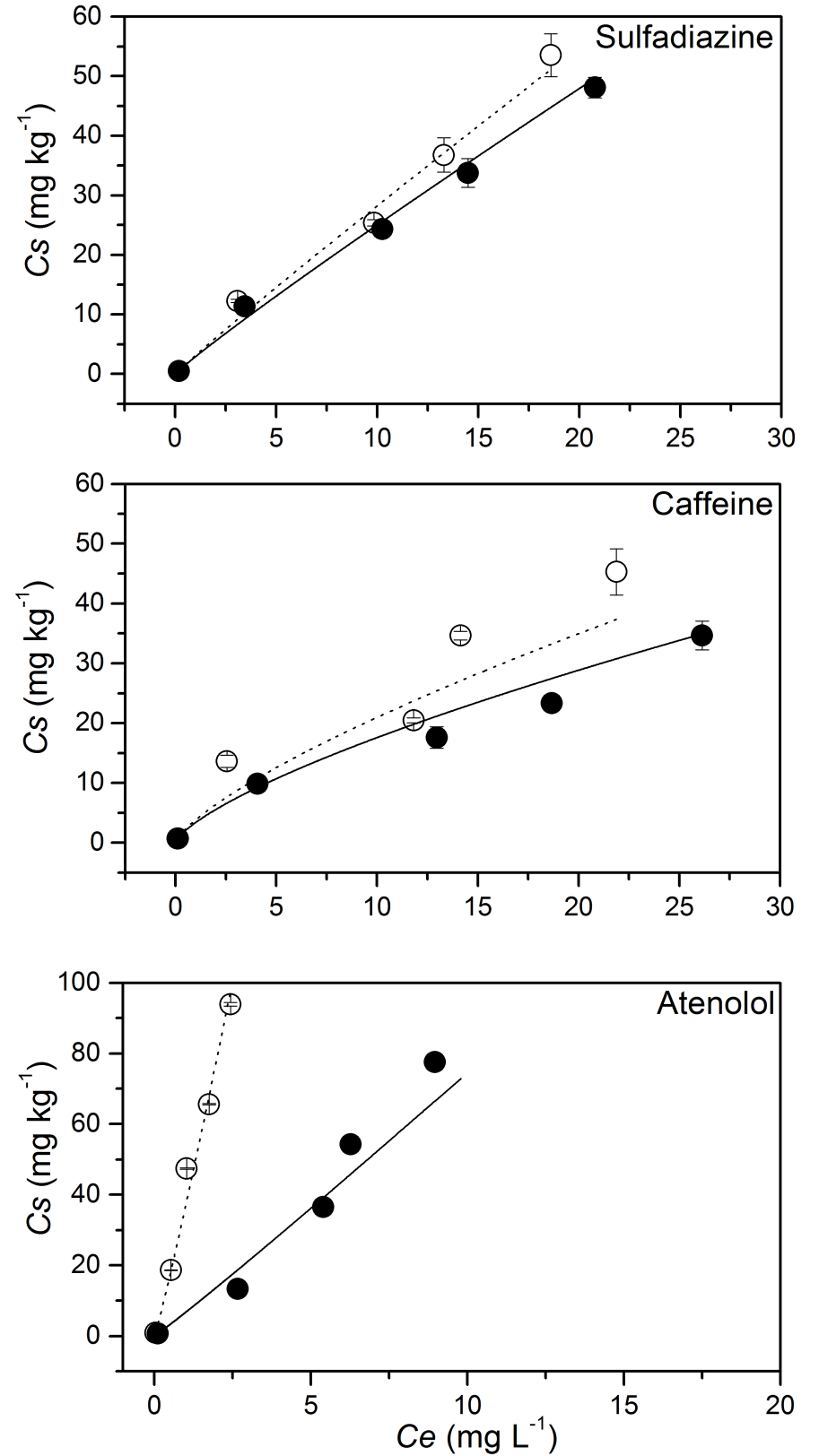


**Fig. S2** Freundlich sorption isotherms of sulfadiazine, caffeine and atenolol in soil I in the presence (filled circles) and absence (open circles) of manure DOM; lines are curve fits using the Freundlich equation; error bars indicate standard errors of three replicate samples (bars not shown are smaller than the filled symbols)


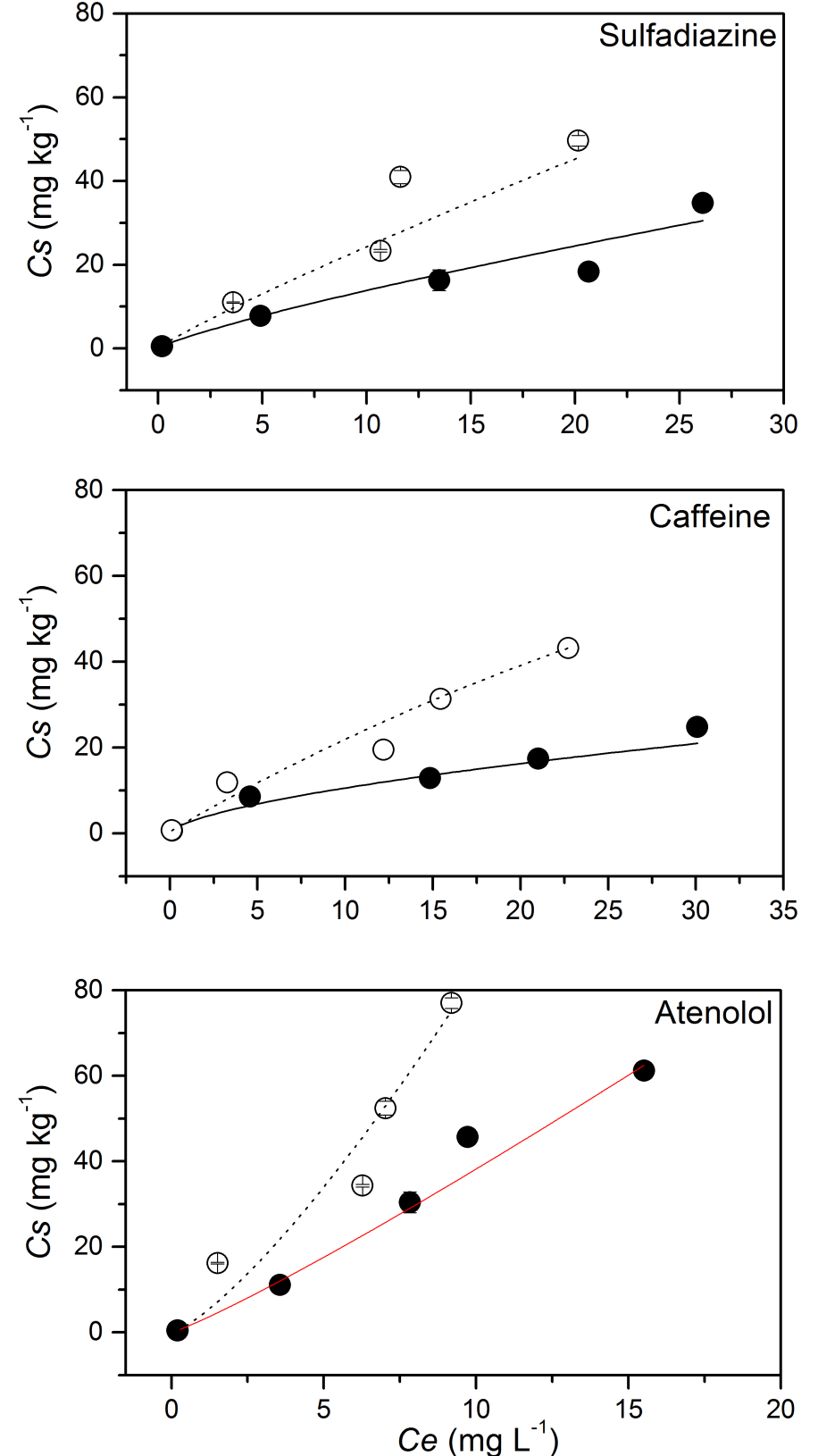


**Fig. S3** Freundlich sorption isotherms of sulfadiazine, caffeine and atenolol in soil II in the presence (filled circles) and absence (open circles) of manure DOM; lines are curve fits using the Freundlich equation; error bars indicate standard errors of three replicate samples (bars not shown are smaller than the filled symbols)


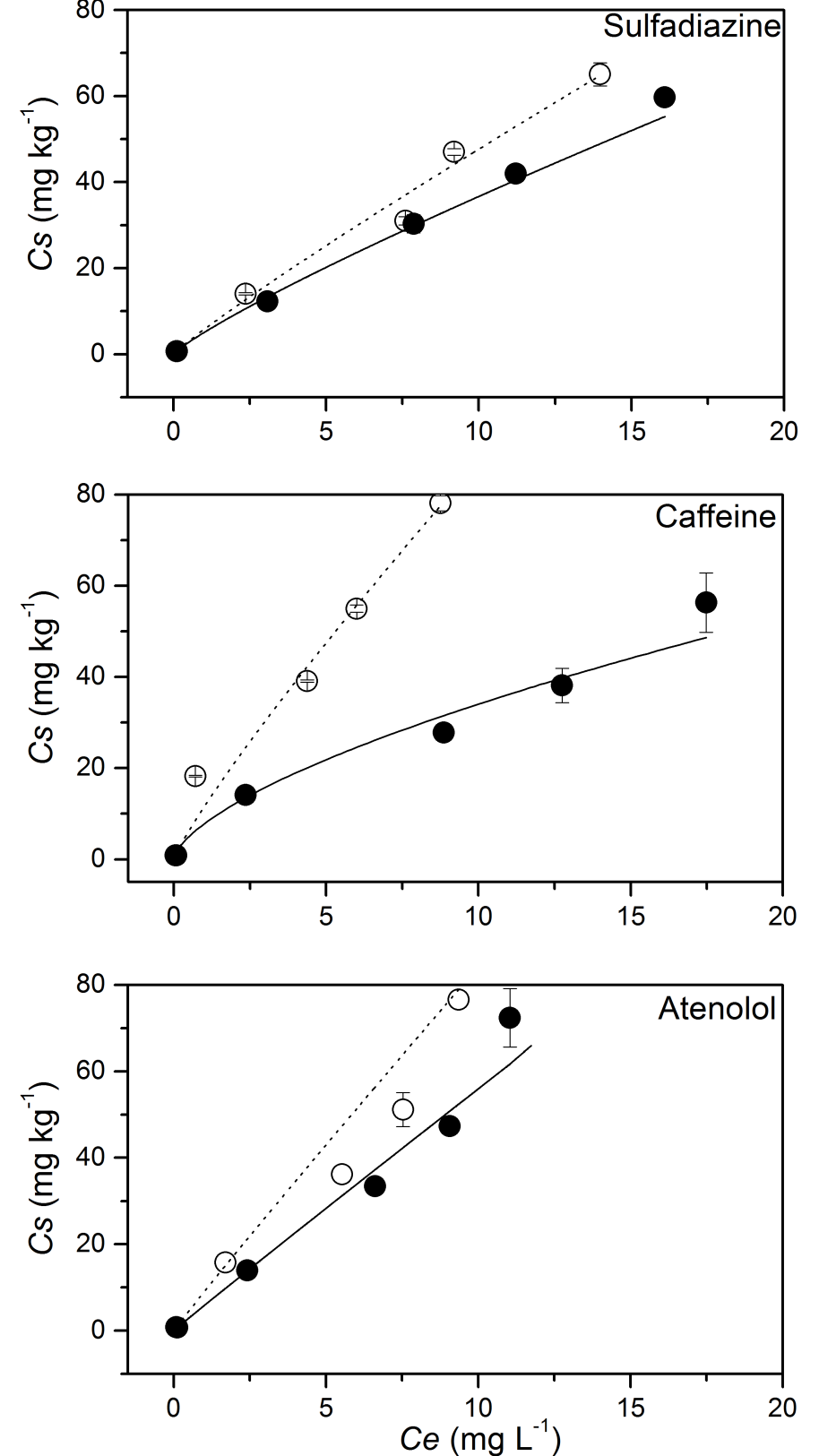


**Fig. S4** Freundlich sorption isotherms of sulfadiazine, caffeine and atenolol in soil IV in the presence (filled circles) and absence (open circles) of manure DOM; lines are curve fits using the Freundlich equation; error bars indicate standard errors of three replicate samples (bars not shown are smaller than the filled symbols)


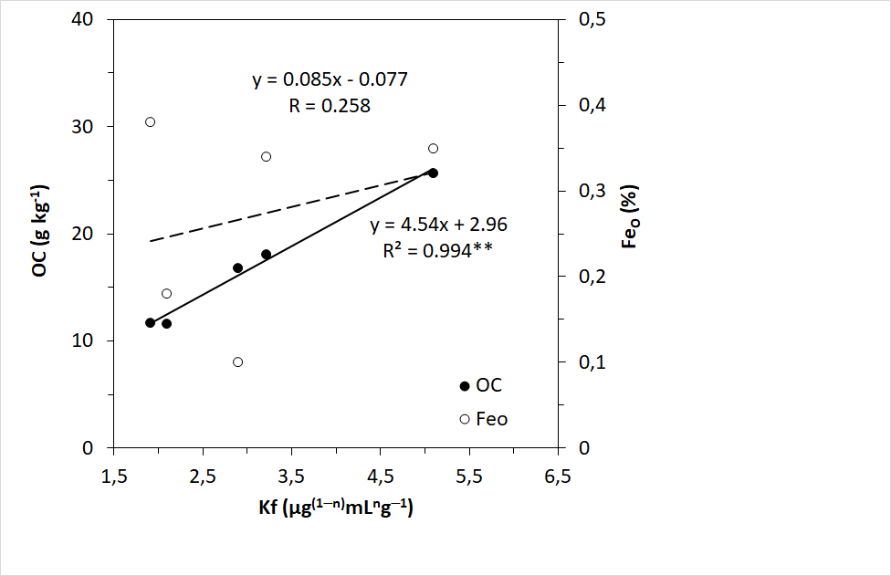


R = 0.994**

R = 0.763*

**with manure DOM**

**without manure DOM**

**(a)**

**(b)**

**Fe_O_ (%)**

0.5

0.4

0.3

0.2

0.1

0

2 3 4 5 6

***K_f_* (μg^(1−n)^ mL^n^ g^−1^)**

**Fe_O_ (%)**

2 3 4 5 6

***K_f_* (μg^(1−n)^ mL^n^ g^−1^)**

0.5

0.4

0.3

0.2

0.1

0


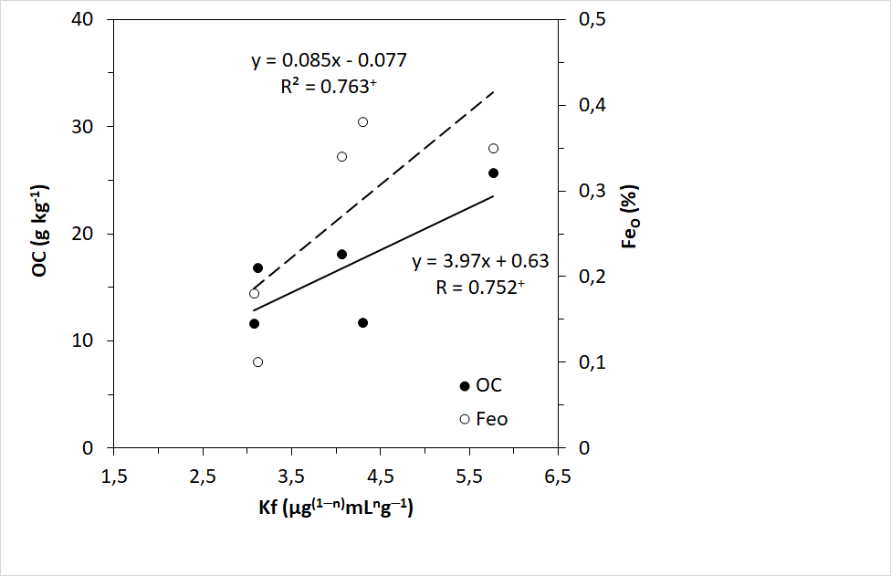


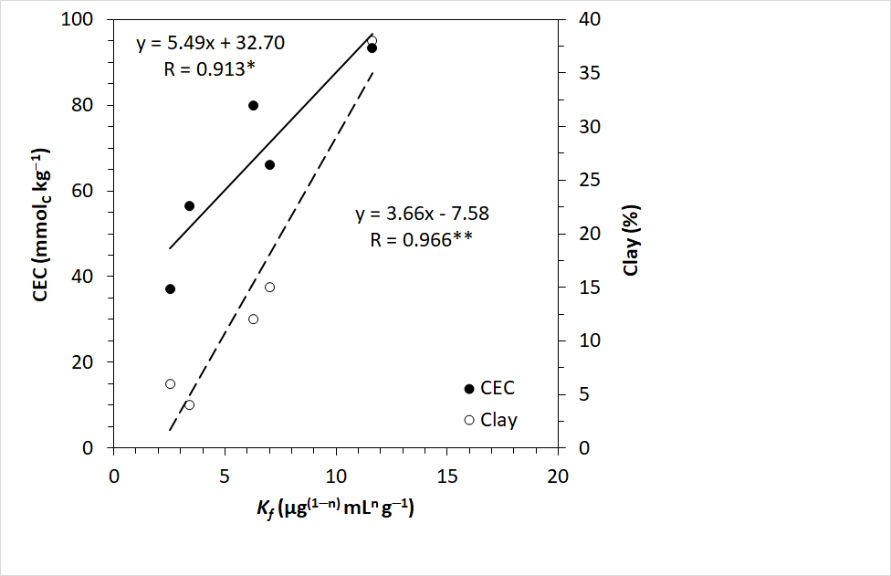


**(c)**

**(d)**


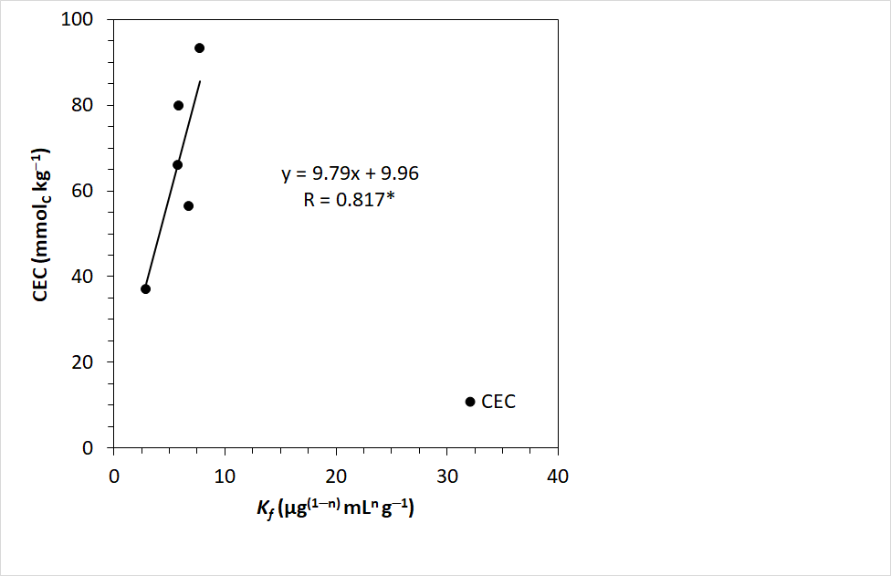

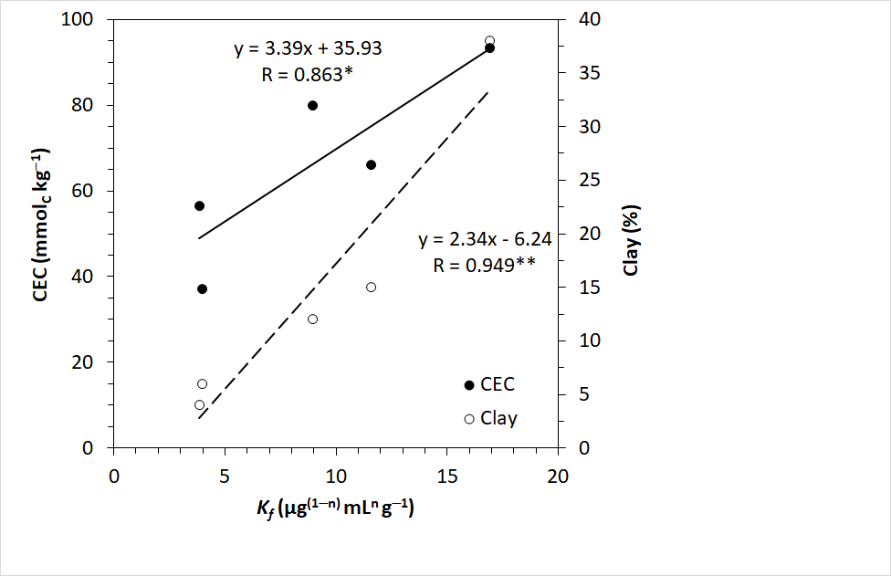


**(f)**

**(e)**


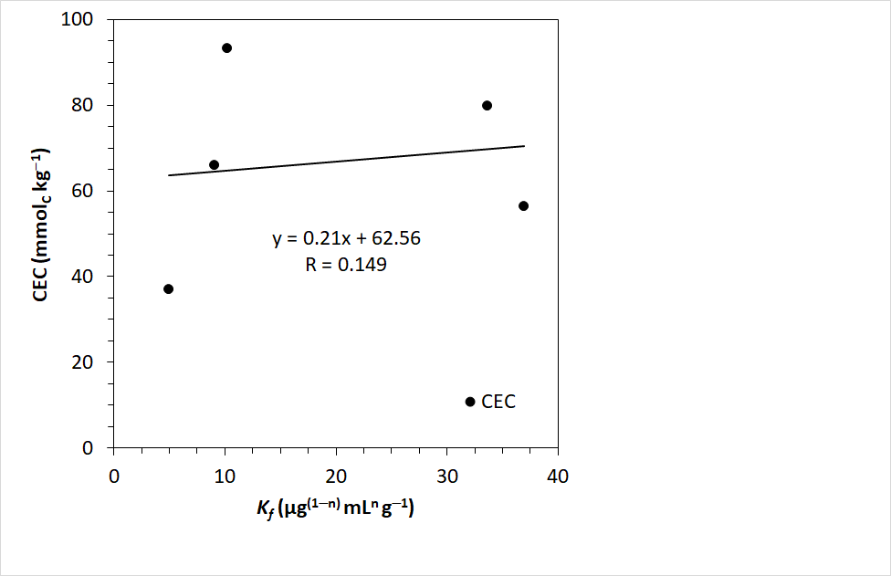


**Fig. S5** Correlations of the Freundlich sorption coefficients (*K_f_*) of (a, b) sulfadiazine, (c, d) caffeine and (e, f) atenolol to different properties of the five tested soils. Correlations found for soil sorption in the absence (left panels) and presence (right panels) of manure DOM; lines are curve fits using the linear equation
